# Dietary Preferences and Sarcopenia in Young and Middle-Aged Adults: A Population-Based Correlational Study

**DOI:** 10.3390/nu18040570

**Published:** 2026-02-09

**Authors:** Wenwen Du, Wen Xu, Minxia Lu, Ming Zhou, Lifeng Tan

**Affiliations:** 1 Department of Nutrition and Food Hygiene, School of Public Health, Nanjing Medical University, Nanjing 211166, China; dwenwen915@163.com (W.D.); 15158886369@163.com (W.X.); 2 Department of Out-Patient, Changzhou Center for Disease Control and Prevention, Changzhou 213003, China; kongmy3401@163.com; 3 Laboratory for Digital Intelligence & Health Governance, Nanjing Medical University, Nanjing 211166, China; 4 Changzhou Institute for Advanced Study of Public Health, Nanjing Medical University, Changzhou 213003, China

**Keywords:** dietary preferences, sarcopenia, muscle strength, young and middle-aged adults

## Abstract

**Background/Objectives**: Sarcopenia, characterized by loss of muscle strength and mass, is a growing health concern. Identifying modifiable risk factors, such as diet, in early adulthood is crucial for prevention. This study aimed to investigate the association between dietary preference patterns and sarcopenia-related indicators in young and middle-aged adults. **Methods**: In this cross-sectional study, 608 participants (median age: 34.0 years, IQR: 24.0–41.0 years) were included. Dietary preferences were assessed using a validated food preference questionnaire, and dietary preference patterns were derived via principal component analysis. The derived pattern scores were standardized for use in subsequent logistic regression models. Handgrip strength (HGS) was measured using a dynamometer, and appendicular skeletal muscle mass index (ASMI) was assessed via bioelectrical impedance analysis. Multivariate logistic regression was used as the primary analysis to evaluate associations, followed by exploratory subgroup analyses. **Results**: Six dietary preference patterns were identified. After adjusting for confounders, the “Ultra-Processed Food Preference” pattern was significantly associated with low handgrip strength (OR = 1.302, 95% CI: 1.072, 1.581). This association was more pronounced in subgroups with medium-to-low income (interaction *p* < 0.05). No significant associations were found between any dietary preference pattern and low ASMI. **Conclusions**: A preference for Ultra-Processed Food is independently associated with lower muscle strength in young and middle-aged adults, with the risk concentrated in specific sociodemographic and behavioral subgroups. These findings highlight the potential importance of addressing unhealthy snack choices early in life in the context of muscle health preservation strategies.

## 1. Introduction

The global population is experiencing a marked aging trend, which presents a major public health challenge [1]. Sarcopenia, an age-related syndrome characterized by the progressive loss of skeletal muscle mass and strength, is a primary cause of functional decline, disability, and mortality in older adults [2]. Projections indicate a dramatic rise in its prevalence, with the global number of affected individuals expected to increase from 50 million to 200 million in the coming decades [3].

The traditional view of sarcopenia as a condition exclusive to old age is being revised [3]. Current research emphasizes its life-course nature, recognizing that risk factors can emerge in early life stages [4,5,6]. Furthermore, multiple cohort studies confirm that its pathological progression does not commence solely in old age. The health gap between the development of sarcopenia and the maintenance of muscle function during normal aging manifests and accumulates from early life, establishing a latent risk for its onset in adulthood and later years [7]. Muscle mass and strength exhibit dynamic changes across the lifespan, typically peaking in young and middle adulthood before gradually declining in old age [8,9]. Research indicates that peak muscle mass and strength are generally attained between the ages of 30 and 40, establishing this period as a critical window for preventive interventions [10,11].

Nutrition and lifestyle play pivotal roles in the development and prevention of sarcopenia. Factors such as smoking and abnormal sleep duration are associated with an elevated risk [12,13], while evidence for alcohol consumption remains inconclusive [14]. The research paradigm in this field is shifting from a focus on isolated nutrients to a comprehensive evaluation of overall dietary patterns [15,16]. Currently, studies investigating the diet-sarcopenia relationship predominantly employ methods like the Food Frequency Questionnaire (FFQ) or dietary records to assess population-level dietary intake. These methods, by systematically collecting data on the frequency and quantity of various food consumptions, can effectively reflect dietary patterns and provide a data foundation for analyzing their association with sarcopenia. However, traditional dietary assessment tools like the FFQ are limited by recall bias and often fail to accurately represent long-term dietary habits [17]. Individual food preferences, being relatively stable and predictive of actual intake, offer a promising alternative for assessing habitual dietary exposure. Evidence suggests that food preference is a predictor of dietary intake and may serve as a reliable alternative to FFQ for evaluating population dietary status [18,19,20,21]. It should be noted, however, that self-reported preferences can be influenced by socioeconomic, cultural, or environmental factors (e.g., food accessibility, cost). Therefore, this study focuses on young and middle-aged adults. It aims to identify predominant dietary preference patterns within this population using Principal Component Analysis and to systematically examine the associations between these patterns and key indicators of sarcopenia, particularly muscle strength and muscle mass. The findings are expected to provide valuable epidemiological evidence for formulating early nutritional strategies to prevent sarcopenia.

## 2. Materials and Methods

### 2.1. Study Population

Participants were recruited from the physical examination centers of the Affiliated Sir Run Run Hospital of Nanjing Medical University and the Changzhou Center for Disease Control and Prevention in Jiangsu Province. All participants provided written informed consent, and the study protocol was approved by the Ethics Committee of Nanjing Medical University (Approval No.: 2023-518). Inclusion criteria were as follows: aged 18–60 years; normal cognitive and physical function with no known impairments affecting motor or metabolic performance; and good compliance to complete all required assessments. Exclusion criteria included: postmenopausal women (owing to the significant confounding effects of menopausal status on muscle physiology and metabolic profiles); a history of musculoskeletal disorders (e.g., active arthritis, severe osteoporosis), fractures, or major joint surgery within the preceding 6 months; presence of neuromuscular disorders; a diagnosis of metabolic or endocrine diseases (e.g., diabetes mellitus) or severe cardiac, cerebral, hepatic, or renal insufficiency; long-term use of medications that interfere with muscle metabolism (e.g., glucocorticoids, androgen/growth hormone preparations, diuretics); and the presence of electronic implants or metallic prostheses within the body [22,23,24].

### 2.2. Questionnaire Survey

Each participant was invited to complete a structured questionnaire designed to collect comprehensive data. The questionnaire included: (1) demographic characteristics (e.g., age, body mass index, gender, education, marital status, per capita annual household income, ethnicity, and religious belief); (2) lifestyle factors (e.g., sleep duration, smoking and drinking, food allergy, and vegetarian practice); and (3) use of dietary supplements (e.g., vitamin D and calcium). Vitamin supplementation was recorded if the consumed multivitamin contained the specific vitamin. A current smoker was defined as an individual who had smoked continuously or cumulatively for at least 6 months and had smoked within the past month. A current drinker was defined as an individual who consumed alcohol at least once per week during the past month. Vegetarian diets were categorized as: vegan (consuming no animal products), pescatarian with egg consumption (consuming fish and seafood along with plant-based foods but no other meat), lacto-ovo vegetarian (consuming dairy and eggs but no meat or fish), and non-vegetarian (consuming a mixed diet of animal and plant foods).

### 2.3. Physical Activity Intensity

The level of physical activity was assessed using the short form of the International Physical Activity Questionnaire (IPAQ-SF) [25]. This internationally validated standardized instrument, characterized by good reliability and validity, is widely used in physical activity surveys among adult populations. Participants retrospectively reported their physical activity over the past 7 days. The IPAQ-SF scoring system employs metabolic equivalent (MET) values, with specific assignments: 8 METs for vigorous-intensity activity, 4 METs for moderate-intensity activity, and 3.3 METs for walking. The total score was calculated by summing the MET-minutes/week for each activity category using the formula: MET value × days per week (d/w) × minutes per day (min/d). Based on the weekly activity frequency and total MET-minutes, participants were subsequently classified into high, moderate, or low physical activity levels.

### 2.4. Dietary Preference Score

The food preference questionnaire for this study was developed based on existing dietary assessment tools, incorporating the dietary habits of the Chinese population. The questionnaire collected information on the degree of preference for 6 major food categories (including 58 subcategories), such as cereals (e.g., millet, rice, corn), tubers (e.g., potato, sweet potato), legumes (e.g., soybean, red bean, mung bean), mushrooms and algae (e.g., mushroom, kelp, tremella), vegetables (leafy, stem, root, gourd, allium, legume pods, solanaceous, and pickled vegetables), fruits (berries, melons, drupes, citrus, pomes), nuts and seeds (e.g., walnut, peanut, chestnut), livestock and poultry meat (e.g., pork, beef, lamb, poultry, offal, processed meat), dairy products (e.g., milk, yogurt, cheese), eggs (e.g., chicken egg, duck egg, salted egg, preserved egg), fish and seafood (e.g., saltwater fish, freshwater fish, shrimp, crab, mollusks), and beverages (e.g., carbonated drinks, fruit/vegetable juices, milk-containing beverages, coffee, tea). Preference for each item was rated using a 5-point Likert scale (0 = never consumed, 1 = strongly dislike, 2 = dislike, 3 = neutral, 4 = like, 5 = strongly like). Dietary preference patterns were derived via principal component analysis. Individual adherence to each pattern was represented by factor scores, which were calculated using the regression method and automatically saved as standardized variables (mean = 0, SD = 1). Six distinct patterns were extracted, collectively explaining 35% of the total variance in the dietary data. Components were extracted based on the Kaiser-Guttman criterion (eigenvalue > 1) and scree plot analysis. To obtain a simpler and more interpretable structure, orthogonal rotation was performed using the Varimax method. Following rotation, six principal components were retained. For each component, food items with absolute factor loadings greater than 0.51 were considered significant contributors. This threshold was chosen to ensure clear delineation of the patterns, as it helped to minimize cross-loadings and assign each food item unambiguously to a single, dominant pattern, thereby enhancing interpretability.

### 2.5. Principal Component Analysis

The reliability of the questionnaire was assessed using Cronbach’s Alpha coefficient. The overall scale demonstrated a Cronbach’s Alpha of 0.929, which exceeds the commonly accepted threshold of 0.9, indicating excellent internal consistency reliability. To reduce multicollinearity among food preference variables and identify underlying dietary preference patterns, principal component analysis (PCA) was performed on the preference scores of the 58 food items. Prior to PCA, the suitability of the data for factor analysis was assessed. The Kaiser–Meyer–Olkin (KMO) measure of sampling adequacy was 0.908, substantially exceeding the acceptable threshold of 0.7. Bartlett’s test of sphericity was significant (approximate χ^2^ = 14285.669, *df* = 1653, *p* < 0.001), indicating sufficient correlations among variables for PCA.

### 2.6. Measurement of Skeletal Muscle Index and Handgrip Strength

Body composition was measured using a bioelectrical impedance analysis (BIA) device (MC-780MA, TANITA, Tokyo, Japan). All bioelectrical impedance analyses were conducted under standardized conditions, including requiring participants to fast before testing, avoid intense exercise, and schedule measurements in the morning to minimize the impact of factors such as hydration status on the test results. Participants were instructed to remove outerwear and metal objects, stand barefoot on the instrument, and follow prompts to place their feet on the electrodes and hold the handgrips to complete the assessment. Data including body weight, body fat percentage, and muscle mass were obtained. The appendicular skeletal muscle mass index (ASMI) was subsequently calculated. According to the latest diagnostic criteria from the Asian Working Group for Sarcopenia (AWGS 2025), low ASMI was defined as <7.6 kg/m^2^ for men and <5.7 kg/m^2^ for women [26].

Maximum handgrip strength (HGS) of the dominant hand was measured using a calibrated electronic hand dynamometer (Xiangshan EH201 R, Zhongshan, China, range: 5–100 kg). Participants stood upright with their arms hanging naturally at their sides. The grip span was adjusted so that the second knuckle of the index finger formed an angle of approximately 90 degrees. Participants were then instructed to squeeze the handle with maximal force. After each attempt, a rest period of at least 20 s was provided. The measurement was repeated at least twice. If the difference between two results exceeded 2.5 kg, a third measurement was taken. The average of valid measurements was recorded as the maximum handgrip strength. Low HGS was defined as <34 kg for men and <20 kg for women [26].

### 2.7. Statistical Analysis

Statistical analyses were performed using SPSS (version 27) and R software (version 4.3.3). Continuous variables with a normal distribution are presented as mean ± standard deviation, while non-normally distributed variables are presented as median and interquartile range. Categorical variables are expressed as percentages (%). The Mann–Whitney U test and the Chi-square test were used for group comparisons of non-normally distributed continuous variables and categorical variables, respectively. The validity and reliability of the dietary preference questionnaire were examined using reliability analysis and factor analysis. Dietary preference patterns were derived from population food preference data via principal component analysis. Multivariate logistic regression analysis was employed to assess the associations between dietary preference patterns and the outcome variables (ASMI and HGS), adjusting for potential confounding factors. Subgroup analyses were further conducted. For the regression models, categorical covariates were coded as dummy variables. The variance inflation factor (VIF) was used to assess multicollinearity, and all VIF values were below 10, indicating no substantial multicollinearity concerns. These subgroup and interaction analyses were exploratory in nature, aimed at generating hypotheses regarding potential effect modification by sociodemographic and behavioral factors. For all analyses, a two-sided *p*-value < 0.05 was considered statistically significant.

## 3. Results

### 3.1. Baseline Characteristics of the Study Population

The study included a total of 608 participants. Among them, 349 individuals (57.40%) had normal HGS, while 259 individuals (42.60%) had low HGS. Statistically significant differences (*p* < 0.05) were observed between the two groups for BMI, education level, and physical activity level. No significant differences (*p* > 0.05) were found for the continuous age variable, gender, per capita annual household income, marital status, ethnicity, religious belief, smoking, drinking, sleep duration, food allergy, vegetarian practice, vitamin D supplementation, or calcium supplementation.

Regarding skeletal muscle mass, 545 participants (89.64%) had a normal ASMI, and 63 (10.36%) had a low ASMI. Significant differences (*p* < 0.05) between these groups were found for BMI and gender. However, no significant differences (*p* > 0.05) were observed for age, per capita annual household income, marital status, education level, ethnicity, religious belief, smoking, drinking, daily sleep duration, food allergy, vegetarian practice, vitamin D supplementation, calcium supplementation, or physical activity level (Table 1).

### 3.2. Dietary Preference Patterns Derived by PCA

Table 2 presents the food items included in the principal component analysis (PCA) and their rotated factor loadings for each identified dietary preferences pattern. The patterns were subsequently named and interpreted based on the food items with high absolute loadings, as follows: Pattern 1: Diversified Vegetable Preference (characterized by leafy, root, and gourd vegetables); Pattern 2: Ultra-Processed Food Preference (characterized by chocolate/candy, pastries/biscuits, and chips/french fries); Pattern 3: Whole Grain & Coarse Cereal Preference (characterized by black rice, millet, and oats); Pattern 4: Diversified Fruit Preference (characterized by various fruits); Pattern 5: Aquatic & Seafood Preference (characterized by saltwater and freshwater fish); and Pattern 6: Livestock & Poultry Meat Preference (characterized by pork, beef and poultry).

### 3.3. Associations of Dietary Patterns with Low HGS and Low ASMI in Multivariable Logistic Regression

Multivariate logistic regression analyses revealed that, among all six identified dietary preference patterns, only the “Ultra-Processed Food Preference” was significantly associated with low HGS in the fully adjusted model (Model 3: OR = 1.302, 95% CI: 1.072–1.581, *p* = 0.008). No other dietary preference patterns showed a significant association with low HGS, and none of the six dietary preference patterns were associated with low ASMI (Table 3).

### 3.4. Subgroup Analysis

**Figure 1:**
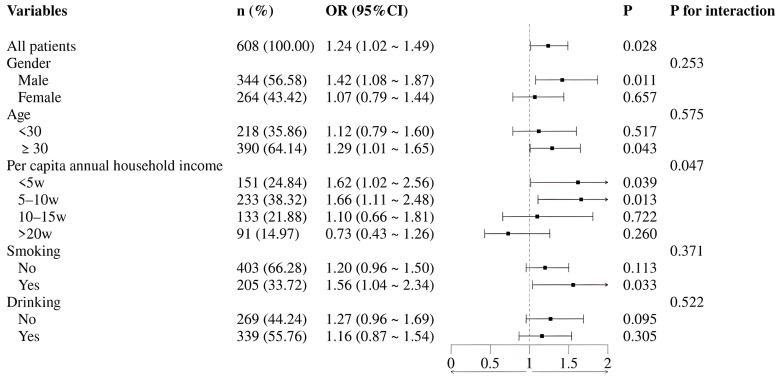
Forest plot of subgroup analyses for the association between Ultra-Processed Food Preference and low handgrip strength. Subgroup-specific odds ratios and 95% confidence intervals were derived from the fully adjusted model (Model 3). The vertical line represents an odds ratio (OR) of 1. An OR > 1 indicates increased risk of low handgrip strength associated with the dietary preference.

To further explore potential population differences in the association between a Ultra-Processed Food Preference and low HGS, subgroup analyses were conducted. The results revealed a significant interaction by per capita annual household income (*p* for interaction = 0.047). Specifically, this detrimental association was statistically significant among individuals with low income (OR = 1.62, 95% CI: 1.02–2.56) and medium income (OR = 1.66, 95% CI: 1.11–2.48), but not among those with high income. Furthermore, significant associations were also observed in males (OR = 1.42, 95% CI: 1.08–1.87), individuals over 30 years of age (OR = 1.29, 95% CI: 1.01–1.65), and current smokers (OR = 1.56, 95% CI: 1.04–2.34) (Figure 1).

## 4. Discussion

This cross-sectional study preliminarily explored the associations between dietary preference patterns and sarcopenia-related indicators (handgrip strength and the skeletal muscle mass index) among young and middle-aged adults. The main findings can be summarized as follows. First, after adjusting for multiple confounders, an “Ultra-Processed Food Preference” was independently associated with low HGS. This suggests that a higher preference for such snacks is correlated with lower muscle strength in this population. Second, a significant interaction was observed for socioeconomic status, with the association being more pronounced in the low-to-medium income subgroup. Differences in the strength of the association were also noted across other subgroups (e.g., by sex, age, and smoking status), although formal tests for interaction were not statistically significant for these factors. Finally, no significant associations were observed between other identified dietary preference patterns and ASMI in this study.

In this study, a preference for an “Ultra-Processed Food Preference” pattern was significantly associated with decreased handgrip strength. This observation is corroborated by a recent prospective cohort study in middle-aged and older Chinese adults, which found that higher ultra-processed food intake was significantly associated with grip strength decline and an increased risk of developing low muscle strength [27]. Higher preference for these foods is associated with biological processes, such as systemic inflammation and oxidative stress, that are theorized to contribute to degenerative changes in muscle tissue and may be linked to the development of sarcopenia [28]. A potential explanatory mechanism involves macronutrient imbalance. Diets rich in fat but low in carbohydrate density have been linked to an increased risk of declining physical function in older adults [29]. Furthermore, certain by-products generated during food processing, such as preservatives and advanced glycation end products, have been correlated with adverse changes in muscle health indicators in adults [30]. Our study focused on a young and middle-aged cohort, suggesting that adverse lifestyle factors may be relevant to muscle function well before clinical diagnosis. This aligns with the life-course approach to muscle health emphasized in the updated AWGS 2025 consensus, which advocates for proactive attention to starting earlier in life [26]. Thus, dietary habits in early adulthood may be an important focus for future strategies aimed at preserving long-term muscle functional reserve.

Notably, exploratory subgroup analyses revealed an unequal distribution of this detrimental association. It was significantly stronger among individuals with a medium to low annual household income (interaction *p* = 0.047), suggesting a potential modifying role for socioeconomic factors [31,32,33]. Under economic constraints, energy-dense, nutrient-poor processed foods often represent a more economical choice, while access to healthier food options may be limited. The association also appeared stronger among males, individuals over 30, and current smokers, which may be related to differences in exposure levels, age-related metabolic changes, and the coexistence of risk behaviors. However, it is important to note that the formal tests for interaction by age and sex were not statistically significant. Therefore, while these patterns are suggestive, they should be interpreted with caution and require replication in larger studies specifically powered to detect such interactions. Collectively, these exploratory findings propose the hypothesis that the link between Ultra-Processed Food Preference and lower muscle function may be moderated by a complex interplay of socioeconomic context, life stage, and behavioral patterns. This association in individuals over 30 years old deserves specific discussion, though no significant linear age effect was observed in our main models. This finding suggests that age may act as an effect modifier in the relationship between Ultra-Processed Food Preference and HGS. This is consistent with the life-course perspective, which holds that early adulthood is a critical window for establishing dietary habits, and these habits are related to functional health outcomes in later life.

In addition, no significant association was observed between dietary preference patterns and low ASMI in this study. Several factors may contribute to this null finding. First, the bioelectrical impedance analysis (BIA) used here has limited sensitivity to early and subtle changes in muscle mass, which may not capture minor variations in younger adults. Second, declines in muscle function often precede measurable loss of muscle mass; in a young cohort, dietary influences may manifest earlier in muscle function rather than in muscle volume. Third, the relatively small number of participants with low ASMI also limited the statistical power to detect significant associations. Together, these considerations suggest that null findings regarding muscle mass measured by BIA in relation to diet should be interpreted cautiously in younger populations. Furthermore, our dietary preference measure did not capture total energy intake or the quantity and quality of protein intake, which are established determinants of muscle mass maintenance. The absence of these variables represents an unmeasured source of residual confounding that may partly explain the lack of observed association between dietary patterns and ASMI in this study.

Finally, the participants in our study were recruited from physical examination centers, which may lead to “healthy participant bias.” People who have regular physical examinations are usually more concerned about their health, and their overall health status, health behaviors, and socioeconomic conditions are better than those in the general community. This bias may underestimate the real association between dietary preferences and muscle health markers, especially for relatively stable indicators such as ASMI. The reason is that there may be restrictions on the range of these outcome indicators. In addition, the generalization of our research results to more vulnerable groups is limited. These groups include people with low healthcare utilization or poor socioeconomic status, who may have a higher risk of poor muscle health. Therefore, future studies should be conducted in more representative community-based cohorts to verify and expand these findings.

A key strength of this study is its focus on young and middle-aged adults, a population underrepresented in sarcopenia research. Another advantage is the use of principal component analysis to derive dietary preference patterns reflective of local food culture. This approach more accurately captures real-world eating behaviors compared to analyses based on single nutrients or foods. Furthermore, subgroup analyses provided preliminary insights into potential population differences in these associations.

However, several limitations should be acknowledged. First, the cross-sectional design precludes causal inference. Reverse causality may exist: for instance, individuals with lower muscle strength may be more inclined to select and prefer more convenient, high-energy ultra-processed foods due to their lifestyle factors, occupational requirements, or underlying health conditions. In addition, there are confounding factors that were not accounted for in the present study. Second, the null finding for ASMI likely stems from limited statistical power (n = 63 cases) coupled with methodological factors, including the reduced sensitivity of BIA for detecting early muscle changes. Thus, this result requires cautious interpretation and validation in studies with more precise measures. Third, our dietary preference patterns reflect self-reported preferences, not measured intake. Preferences can be influenced by socioeconomic and cultural factors and may not equate to actual consumption. This should be considered when interpreting the associations, as they link dietary inclinations to muscle health, not specific nutrient intakes. Fourth, while the exclusion criteria (e.g., postmenopausal women and individuals with common metabolic diseases) were implemented to control for key confounding factors (such as the profound impact of menopausal hormonal changes on muscle metabolism) and strengthen internal validity, they limit the generalizability of the findings. Therefore, the conclusions of this study are mainly applicable to relatively healthy young and middle-aged adults, and caution should be exercised when extrapolating the conclusions to a broader adult population, especially elderly women and patients with chronic diseases. Fifth, the examination of multiple dietary preference patterns, outcomes, and exploratory subgroup analyses increases the risk of type I error (false-positive findings).

## 5. Conclusions

This study indicates that a “Ultra-Processed Food Preference” is associated with decreased muscle strength in young and middle-aged adults. This detrimental association was moderated by lower socioeconomic status. These findings identify a hypothesis and a potential risk marker for future investigation. Further prospective studies are warranted to establish the causal relationship between dietary preference patterns and changes in muscle health.

## Figures and Tables

**Table 1:** Baseline characteristics of participants stratified by HGS and ASMI status.

Variables	Normal HGS (n = 349)	Low HGS (n = 259)	*p*-Value	Normal ASMI (n = 545)	Low ASMI (n = 63)	*p*-Value
Age (years)	34.00 (25.00, 40.00)	32.00 (22.00, 42.00)	0.348	34.00 (24.00, 41.00)	30.00 (23.00, 39.00)	0.261
BMI (kg/m^2^)	23.80 (21.40, 26.80)	22.40 (19.90, 25.30)	<0.001	23.80 (21.40, 26.80)	19.60 (18.05, 20.85)	<0.001
Gender			0.056			0.025
Male	209 (60.76)	135 (39.24)		300 (87.21)	44 (12.79)	
Female	140 (53.03)	124 (46.97)		245 (92.80)	19 (7.20)	
Per capita annual household income			0.163			0.386
<5 w	84 (55.63)	67 (44.37)		140 (92.72)	11 (7.28)	
5–10 w	145 (62.23)	88 (37.77)		208 (89.27)	25 (10.73)	
10–15 w	67 (50.38)	66 (49.62)		115 (86.47)	18 (13.53)	
>15 w	53 (58.24)	38 (41.76)		82 (90.11)	9 (9.89)	
Marital status			0.137			0.124
Never married	130 (53.06)	115 (46.94)		213 (86.94)	32 (13.06)	
Married	205 (59.77)	138 (40.23)		315 (91.84)	28 (8.16)	
Divorced	14 (70.00)	6 (30.00)		17 (85.00)	3 (15.00)	
Education			0.029			0.324
High school or below	127 (58.26)	91 (41.74)		200 (91.74)	18 (8.26)	
College	70 (67.96)	33 (32.04)		89 (86.41)	14 (13.59)	
Bachelor’s degree or above	152 (52.96)	135 (47.04)		256 (89.20)	31 (10.80)	
Ethnicity			0.433			0.121
Han Chinese	347 (57.64)	255 (42.36)		541 (89.87)	61 (10.13)	
Other	2 (33.33)	4 (66.67)		4 (66.67)	2 (33.33)	
Religious Belief			0.496			0.542
No	333 (57.12)	250 (42.88)		524 (89.88)	59 (10.12)	
Yes	16 (64.00)	9 (36.00)		24 (96.00)	4 (16.00)	
Smoking			0.106			0.946
No	222 (55.09)	181 (44.91)		361 (89.58)	42 (10.42)	
Yes	127 (61.95)	78 (38.05)		184 (89.76)	21 (10.24)	
Drinking			0.060			0.973
No	143 (53.16)	126 (46.84)		241 (89.59)	28 (10.41)	
Yes	206 (60.77)	133 (39.23)		304 (89.68)	35 (10.32)	
Sleep Duration			0.985			0.640
<6 h	30 (56.60)	23 (43.40)		47 (88.68)	6 (11.32)	
6–8 h	265 (57.36)	197 (42.64)		417 (90.26)	45 (9.74)	
8–10 h	54 (58.06)	39 (41.94)		81 (87.10)	12 (12.90)	
Food Allergy			0.919			0.310
No	324 (57.35)	241 (42.65)		504 (89.20)	61 (10.80)	
Yes	25 (58.14)	18 (41.86)		41 (95.35)	2 (4.65)	
Vegetarian Practice			0.698			0.534
Vegan	2 (50.00)	2 (50.00)		3 (75.00)	1 (25.00)	
Pescatarian with egg consumption	8 (50.00)	8 (50.00)		14 (87.50)	2 (12.50)	
Lacto-ovo vegetarian	9 (47.37)	10 (52.63)		17 (89.47)	2 (10.53)	
Non-vegetarian	330 (58.00)	239 (42.00)		511 (89.81)	58 (10.19)	
Vitamin D Supplementation			0.322			0.286
No	299 (56.63)	229 (43.37)		476 (90.15)	52 (9.85)	
Yes	50 (62.50)	30 (37.50)		69 (86.25)	11 (13.75)	
Calcium Supplementation			0.094			0.553
No	306 (56.25)	238 (43.75)		489 (89.89)	55 (10.11)	
Yes	43 (67.19)	21 (32.81)		56 (87.50)	8 (12.50)	
Physical Activity			0.004			0.611
Low	126 (50.00)	126 (50.00)		223 (88.49)	29 (11.51)	
Moderate	92 (66.19)	47 (33.81)		124 (89.21)	15 (10.79)	
High	131 (60.37)	86 (39.63)		198 (91.24)	19 (8.76)	

Data are presented as median (interquartile range) for continuous variables and number (percentage) for categorical variables. Abbreviations: BMI, body mass index; HGS, handgrip strength; ASMI, appendicular skeletal muscle mass index. *p*-values were derived from the Mann–Whitney U test for continuous variables and the χ^2^ test for categorical variables.

**Table 2:** Rotated factor loadings of food items in dietary preference patterns derived from principal component analysis.

Food Items	Diversified Vegetable Preference	Ultra-Processed Food Preference	Whole Grain & Coarse Cereal Preference	Diversified Fruit Preference	Aquatic & Seafood Preference	Livestock & Poultry Meat Preference
Leafy vegetables	0.731					
Tender stem vegetables	0.629					
Flowering vegetables	0.635					
Root vegetables	0.726					
Gourd vegetables	0.682					
Legume pod vegetables	0.532					
Solanaceous vegetables	0.607					
Chips/French fries		0.733				
Chocolate/Candy		0.81				
Pastries/Biscuits		0.736				
Preserved fruits		0.596				
Black rice			0.782			
Millet			0.705			
Oats			0.714			
Berries				0.671		
Melons				0.724		
Drupes				0.714		
Pomes				0.624		
Saltwater fish					0.718	
Freshwater fish					0.758	
Shrimp					0.554	
Crab					0.635	
Mollusks					0.638	
Pork						0.678
Beef						0.626
Lamb						0.518
Poultry						0.699

**Table 3:** Association between Dietary Preference Pattern Scores and HGS/ASMIs.

	Handgrip Strength OR (95%CI)	*p*-Value	Appendicular Skeletal Muscle Mass OR (95%CI)	*p*-Value
Diversified Vegetable Preference				
Model 1	0.891 (0.756, 1.050)	0.169	0.977 (0.747, 1.278)	0.866
Model 2	0.846 (0.711, 1.007)	0.060	1.058 (0.746, 1.502)	0.751
Model 3	0.865 (0.724, 1.034)	0.111	1.016 (0.725, 1.426)	0.925
Ultra-Processed Food Preference				
Model 1	1.296 (1.094, 1.535)	0.003	1.089 (0.832, 1.424)	0.536
Model 2	1.327 (1.097, 1.607)	0.004	0.855 (0.585, 1.249)	0.417
Model 3	1.302 (1.072, 1.581)	0.008	0.795 (0.521, 1.214)	0.288
Whole Grain & Coarse Cereal Preference				
Model 1	0.941 (0.800, 1.108)	0.468	1.032 (0.793, 1.342)	0.817
Model 2	0.870 (0.730, 1.036)	0.119	0.964 (0.697, 1.334)	0.825
Model 3	0.889 (0.744, 1.063)	0.197	0.910 (0.645, 1.285)	0.593
Diversified Fruit Preference				
Model 1	1.184 (1.005, 1.394)	0.043	1.030 (0.793, 1.337)	0.826
Model 2	1.149 (0.958, 1.378)	0.135	0.955 (0.651, 1.403)	0.816
Model 3	1.150 (0.957, 1.382)	0.136	1.005 (0.685, 1.475)	0.980
Aquatic & Seafood Preference				
Model 1	0.892 (0.756, 1.051)	0.172	0.871 (0.673, 1.126)	0.292
Model 2	0.879 (0.740, 1.044)	0.142	0.897 (0.631, 1.277)	0.548
Model 3	0.899 (0.753, 1.074)	0.242	0.911 (0.627, 1.323)	0.624
Livestock & Poultry Meat Preference				
Model 1	1.008 (0.856, 1.188)	0.922	0.863 (0.661, 1.126)	0.277
Model 2	1.069 (0.901, 1.269)	0.444	0.930 (0.646, 1.337)	0.694
Model 3	1.078 (0.904, 1.285)	0.402	0.923 (0.638, 1.336)	0.671

Model 1: Unadjusted model. Model 2: Adjusted for age, gender, BMI, per capita annual household income, education, marital status, ethnicity, and religious belief. Model 3: Adjusted for variables in Model 2 plus smoking, drinking, sleep duration, food allergy, vegetarian practice, vitamin D supplementation, calcium supplementation, and physical activity.

## Data Availability

The data presented in this study are available on reasonable request from the corresponding author. The data are not publicly available due to privacy and ethical restrictions.
